# Regional adiposity, cardiorespiratory fitness, and left ventricular strain: an analysis from the Dallas Heart Study

**DOI:** 10.1186/s12968-021-00757-w

**Published:** 2021-06-14

**Authors:** Nitin Kondamudi, Neela Thangada, Kershaw V. Patel, Colby Ayers, Alvin Chandra, Jarret D. Berry, Ian J. Neeland, Ambarish Pandey

**Affiliations:** 1 Division of Cardiology, Department of Internal Medicine, University of Texas Southwestern Medical Center, 5323 Harry Hines Blvd, Dallas, TX 75390-9047 USA; 2 Department of Clinical Sciences, University of Texas Southwestern Medical Center, 5323 Harry Hines Blvd, Dallas, TX 75390-9047 USA; 3 Division of Cardiology, Department of Internal Medicine, Northwestern University, Feinberg School of Medicine, Chicago, IL 60611 USA; 4 Division of Cardiology, Department of Internal Medicine, Houston Methodist Hospital, 6550 Fannin St., Houston, TX 77030 USA; 5 Division of Cardiology, Department of Internal Medicine, University Hospitals Harrington Heart and Vascular Institute and Case Western Reserve University School of Medicine, 2103 Cornell Road, Cleveland, OH 44106 USA

**Keywords:** Visceral fat, Lower-body fat, Subcutaneous fat, Left ventricular peak circumferential strain, Heart failure

## Abstract

**Background:**

Low cardiorespiratory fitness (CRF), high body mass index, and excess visceral adiposity are each associated with impairment in left ventricular (LV) peak circumferential strain (E_cc_), an intermediate phenotype that precedes the development of clinical heart failure (HF). However, the association of regional fat distribution and CRF with E_cc_ independent of each other and other potential confounders is not known.

**Methods:**

Participants from the Dallas Heart Study Phase 2 who underwent dual energy X-ray absorptiometry assessment of regional fat distribution, CRF assessment by submaximal treadmill test, and E_cc_ quantification by tissue-tagged cardiovascular magnetic resonance were included in the analysis. The cross-sectional associations of measures of regional adiposity, namely visceral adipose tissue (VAT), subcutaneous adipose tissue (SAT), and lower-body fat (LBF) with E_cc_ after adjustment for CRF and other potential confounders (independent variables) were assessed using multivariable linear regression analysis.

**Results:**

The study included 1089 participants (55% female, 39% black). In the unadjusted analysis, higher VAT was associated with greater impairment in E_cc_. After adjustment for baseline risk factors, CRF, parameters of LV structure and function, and other fat depots such as SAT and LBF, higher VAT remained associated with greater impairment in E_cc_ (β: 0.19, P = 0.002). SAT and LBF were not significantly associated with E_cc_, however, CRF remained associated with E_cc_ in the fully adjusted model including all fat depots (β: − 0.15, P < 0.001).

**Conclusions:**

VAT and CRF are each independently associated with impairment in E_cc_, suggesting that higher VAT burden and low CRF mediate pathological cardiac remodeling through distinct mechanisms.

**Supplementary Information:**

The online version contains supplementary material available at 10.1186/s12968-021-00757-w.

## Introduction

Excess adiposity, as measured by body mass index (BMI), is associated with impaired myocardial function and higher risk of heart failure (HF) [1–6]. However, BMI is an imperfect representation of total adiposity because it does not capture differences in lean body mass and fat distribution. Besides BMI, differences in cardiorespiratory fitness (CRF) and regional fat depots contribute significantly to obesity related HF risk [7, 8]. Although the mechanisms by which CRF and regional fat depots mediate HF risk are not well established, prior studies have reported epidemiological associations between CRF, visceral adiposity (VAT), and abnormal cardiac remodeling patterns. VAT has been associated with higher left ventricular (LV) mass and lower LV end diastolic volume (EDV) [5, 9], while CRF has been associated with higher LV filling pressures and diastolic dysfunction [10, 11]. These cardiac remodeling patterns may reflect intermediate phenotypes in the progression from at-risk to clinical HF [12, 13].

LV peak systolic circumferential strain (E_cc_) is a sensitive marker of abnormal cardiac remodeling and is independently associated with risk of HF [14, 15]. CRF and regional adiposity have each been associated with impairment in E_cc_ independent of BMI [5, 16]. However, prior studies have not examined the association between regional fat distribution and subclinical myocardial dysfunction, independent of CRF, a potential confounder not accounted for in prior reports. Accordingly, we examined the associations between specific adipose tissue depots and E_cc_ while adjusting for cardiovascular disease (CVD) risk factors, CRF, and LV parameters in a multi-ethnic, population-based cohort without known CVD. We hypothesized that higher amounts of VAT would be independently associated with greater subclinical impairment in LV contractility measured by E_cc_.

## Methods

### Study population

The Dallas Heart Study (DHS) is a multiethnic, probability-based, population cohort study of Dallas County residents with deliberate oversampling of black individuals. Methods and study design of DHS have been described previously [17]. Individuals were enrolled in DHS-1 and underwent initial evaluation from 2000 to 2002. Between 2007 and 2009, participants were invited to DHS-2 to complete follow up testing at the University of Texas Southwestern Medical Center that included anthropometric measurements, laboratory tests, imaging studies, and fitness assessments. Patients who underwent fat depot measurements by dual-energy x-ray absorptiometry (DEXA) and cardiovascular magnetic resonance (CMR) were included in the present analysis. CMR was performed in 2106 participants in DHS-2, and 2037 of those studies included tagged CMR of sufficient quality for strain analysis. Participants with CVD, LV ejection fraction (LVEF) < 45%, ß-blocker use, missing CRF data, missing DEXA data, or irregular/sub-optimal maximal predicted heart rate at target workload, were excluded. Heart rates > 100% or < 50% of maximal predicted heart rate at target workload were considered sub-optimal and individuals achieving those levels were also excluded. The final study sample included 1089 participants. All study participants provided written informed consent. The University of Texas Southwestern Medical Center institutional review board has approved the protocol for the DHS Phase II.

### Assessment of regional fat depots and lean body mass

Total body fat and fat-free mass were measured using DEXA (Delphi W scanner, Hologic Inc, Bedford, Massachusetts, USA) as has been previously described [9]. DEXA derived total abdominal subcutaneous fat was subtracted from measured total abdominal fat to estimate VAT mass (total abdominal fat—total abdominal subcutaneous fat = visceral adipose tissue) using APEX software version 13.4.2. Fat below oblique lines crossing the femoral necks to below the pubic symphysis is considered lower-body fat (LBF). Total lean body mass and appendicular lean mass (ALM) were measured by using a dual-beam absorption energy unit (Delphi W, Hologic, Inc.) bone densitometer in array mode. Body composition was quantified using Oasis software (Hologic, Inc.). ALM index was calculated by dividing ALM by height in meters squared.

### Assessment of cardiorespiratory fitness and physical activity levels

A submaximal exercise treadmill test was used to estimate CRF as has been previously described [16, 18]. In brief, oxygen uptake (VO_2_) was estimated using Givoni’s equation for metabolic energy cost. Maximal heart rate was estimated by subtracting the participant age from 220, and then the % maximal heart achieved was calculated. Using Hellerstein’s formula, % maximal heart rate was converted to estimated peak oxygen uptake (VO_2max_) which was used to determine CRF. Moderate to vigorous physical activity (MVPA) levels were assessed among the study participants using an accelerometer (Actical, Philips Respironics, Bend, Oregon, USA) that was worn for 7 consecutive days on their non-dominant wrist. As reported previously, based on the accelerometer output (counts per min [CPM]), MVPA was defined as >  = 1500 CPM. Minutes spent above this threshold were averaged across all valid wear days to estimate the total daily duration of MVPA [19].

### Clinical covariates

Assessment of demographic, clinical, and anthropometric characteristics in the DHS have been described previously [17]. Briefly, race was self‐assigned according to US census categories. Smoking was defined as cigarette use within the previous 30 days and/or a lifetime history of having smoked ≥ 100 cigarettes. Hypertension was defined as blood pressure ≥ 140/90 mm Hg or taking antihypertensive medication(s). Diabetes mellitus was defined as having a fasting serum glucose ≥ 126 mg/dL, self‐reporting a history of diabetes mellitus, or taking hypoglycemic medication. Hypercholesterolemia was defined as a calculated low‐density lipoprotein cholesterol ≥ 160 mg/dL on a fasting sample, direct low‐density lipoprotein cholesterol ≥ 160 mg/dL on a nonfasting sample, total cholesterol ≥ 240 mg/dL, or use of statin medication. BMI was calculated using weight in kilograms divided by height in meters squared. Waist circumference was measured 1 cm superior to the iliac crest.

### Assessment of cardiac structure and function

CMR was performed using a 3 T CMR system (Philips Healthcare, Best, The Netherlands). LV volume and LV mass were quantified using MASS software (Medis Medical Imaging Systems, Leiden, the Netherlands) on short-axis breath-hold electrocardiogram (ECG) gated cine CMR images, as previously described [20]. Stroke volume was calculated as the difference in LV end-diastolic and end-systolic volumes (LVEDV, LVESV). LVEF was calculated using volumes [(LVEDV – LVESV) / LVEDV]*100. E_cc_ was computed using the myocardial tissue tagging method [21, 22]. In brief, myocardium was tagged in the vertical and horizontal planes of the LV mid-ventricular short-axis slices. Strain was measured in six LV wall segments using harmonic phase imaging software offline (HARP, Diagnosoft Virtue 5.04; Diagnosoft, Palo Alto, California, USA). A global circumferential strain (GCS) graph was created with strain values at different LV wall segments and multiple time points throughout the cardiac cycle. The most negative point on the curve reflects E_cc_. Less negative strain represents decreased LV wall shortening circumferentially and suggests impaired contractility, whereas more negative strain reflects more favorable contractility and better cardiac function.

### Statistical analysis

The reproducibility in CMR measures E_cc_ was assessed in a subset of randomly selected participants (n = 30) who had repeat blinded measurements of the Ecc parameter by the same operator on two separate occasions. The correlation between the paired measurements was assessed using a Pearson correlation test. The agreement between the paired measurements was assessed using the Bland Altman plot. Demographic and clinical characteristics of the study population were compared across race- and sex-adjusted quartiles of VAT using Wilcoxon rank-sum test for continuous variables and Chi-square test for categorical variables. The cross-sectional association between regional fat depots, CRF, and Ecc was assessed with separate multivariable adjusted linear regression models. The primary exposure variables were VAT, SAT, and LBF, and the outcome variable was E_cc_. Separate models were constructed for VAT, SAT, and LBF exposure variables with sequential adjustment for the following confounders: Model 1—demographics (age, sex, race/ethnicity), traditional CVD risk factors (smoking status, fasting glucose, diabetes status, hypertension status, systolic blood pressure), and total lean body mass; Model 2—Model 1 + CRF; and Model 3—Model 2 + LV parameters (LV mass index and LVEF). A most adjusted Model 4 was additionally constructed which included all covariates in Model 3 + the three regional adiposity parameters, VAT, SAT, and LBF, in the same model.

Standardized beta estimates were calculated for VAT, SAT, LBF, and CRF in each model, representing relative SD change in E_cc_ per SD change in each respective independent variable while keeping other covariates fixed. Restricted cubic splines of each fat depot and E_cc_ were constructed using Model 3. BMI stratified analysis [obese (BMI > 30 kg/m^2^) vs. non obese] was also performed by evaluating the associations of VAT, SAT, and LBF with E_cc_ among obese participants in the most adjusted model (Model 4). Sensitivity analysis was also performed by constructing additional models replacing total lean body mass with ALM (model 5), ALM index (model 6), and adding MVPA as another covariate to the most adjusted model 4 (model 7). A 2-sided *P* value < 0.05 was considered statistically significant for all hypothesis testing. SAS (version 9.2, SAS Institute, Inc., Cary, North Carolina, USA) was used for statistical analyses.

## Results

A total of 1089 participants (55% female, 39% black, median 48 years, BMI 29.1 kg/m^2^) were studied. In a subset of 30 CMR studies with repeated assessment of E_cc_, we observed a moderate degree of correlation with a Pearson correlation coefficient of 0.542. We also observed adequate agreement on the Bland Altman plot (mean difference: 1.23, limits of agreement: − 2.93 to 5.38, see Additional file 1: Figure S1). Baseline characteristics of the study participants are compared across race- and sex-adjusted quartiles of VAT (Table 1). In cross-sectional analysis, participants with higher amounts of VAT were more likely to be older, less fit, less active, and obese with higher prevalence of CVD risk factors, including hypertension, diabetes, and hypercholesterolemia. Participants with higher amounts of VAT were also more likely to have higher total lean body mass and ALM. Among imaging characteristics, participants with higher VAT were more likely to have greater impairment in E_cc_, lower LVEDV, and higher LVEF. In contrast, LV stroke volume and LV mass index did not differ significantly across quartiles of VAT (Table 1).

**Table 1:** Study population characteristics across race- and gender-adjusted quartiles of visceral adiposity

	Q1	Q2	Q3	Q4	P value
N	268	274	276	271	–
Visceral adipose tissue, kg	1.1 (0.8, 1.4)	1.7 (1.5, 2.5)	2.4 (2.0, 3.3)	3.4 (2.8, 4.3)	–
*Demographics*					
Age, years	45.0 (40.0, 53.5)	48.0 (41.0, 55.0)	51.0 (44.0, 58.0)	53.0 (46.0, 60.0)	< .001
Male, %	45.5	45.6	45.7	45.4	0.978
White, %	42.9	42.7	42.4	42.8	0.963
Black, %	38.8	38.7	38.4	38.8	0.972
Hispanic, %	16.4	16.4	16.7	16.2	0.976
*Vitals*					
Systolic BP, mmHg	120.2 (112.3, 133.3)	124.7 (116.3, 135.0)	126.7(118.0, 140.0)	131.0 (123.0, 139.7)	< .001
MVPA, min/day	53.5 (41.1)	44.4 (35.9)	38.2 (31.3)	34.6 (28.8)	< .001
*Anthropometric measures*					
Body mass index, kg/m^2^	23.9 (21.5, 26.0)	27.6 (25.1, 30.1)	29.7 (27.5, 32.4)	33.8 (31.0, 37.3)	< .001
Waist circumference, cm	81.3 (74.9, 87.0)	88.9 (83.8, 95.3)	96.5 (90.2, 101.6)	106.1 (100.3, 111.8)	< .001
*Medical history*					
Hypertension, %	24.6	28.8	43.5	57.6	< .001
Diabetes mellitus, %	34.7	40.9	38.0	50.6	0.001
Hypercholesterolemia, %	13.9	27.8	25.9	31.2	< .001
Smoking status, %	25.7	21.6	15.5	15.8	0.001
*Laboratory data*					
LDL, mg/dL	111.0 (92.0, 135.0)	120.0 (95.0, 145.0)	119.0 (100.0, 143.0)	116.0 (93.0, 142.0)	0.259
A1C, %	5.3 (5.1, 5.5)	5.4 (5.1, 5.6)	5.5 (5.2, 5.7)	5.6 (5.3, 5.9)	< .001
Estimated peak oxygen uptake (VO_2max_), ml/kg/min	32.9 (25.7, 39.5)	28.4 (21.6, 33.8)	26.8 (21.8, 34.0)	23.9 (18.9, 29.1)	< .001
Body composition					
Total lean body mass, kg	38.3(82.2)	40.4 (86.7)	42.6 (91.9)	45.4 (86.2)	< .001
ALM, kg	21.5 (5.5)	22.2 (5.8)	23.5 (6.1)	24.7 (5.3)	< .001
ALM index, kg/m^2^	7.4 (1.4)	7.7 (1.5)	8.1 (1.5)	8.6 (1.28)	< .001
CMR measures					
LV mass index, g/m^2^	63.1 (54.1, 74.4)	58.9 (50.0, 71.9)	62.1 (51.8, 71.3)	61.6 (54.4, 71.6)	0.632
LVEDV index, mL/m^2^	64.7 (56.8, 71.3)	58.0 (51.8, 66.3)	57.5 (50.8, 63.8)	55.2 (49.1, 61.9)	< .001
LVEF, %	68.3 (64.1, 72.9)	69.4 (65.3, 73.4)	69.1 (65.0, 73.1)	69.6 (65.6, 73.6)	0.043
Stroke volume, mL	78.5 (68.8, 89.8)	76.5 (67.2, 87.4)	78.4 (68.4, 88.6)	80.1 (70.8, 89.2)	0.171
Peak systolic circumferential strain, (%)	− 15.2 (− 17.1, − 13.4)	− 14.8 (− 16.1, − 13.2)	− 14.3 (− 16.3, − 12.3)	− 14.3 (− 16.1, − 12.4)	< .001

Variables expressed in %, median with interquartile range, or mean with standard deviation. *BP* blood pressure, *MVPA* moderate to vigorous physical activity, *LDL* low-density lipoprotein, *ALM* appendicular lean mass, *CMR* cardiovascular magnetic resonance, *LVEF* left ventricular ejection fraction, *LVEDV* left ventricular end diastolic volume

### Association of regional fat depots with peak circumferential strain

**Fig. 1:**
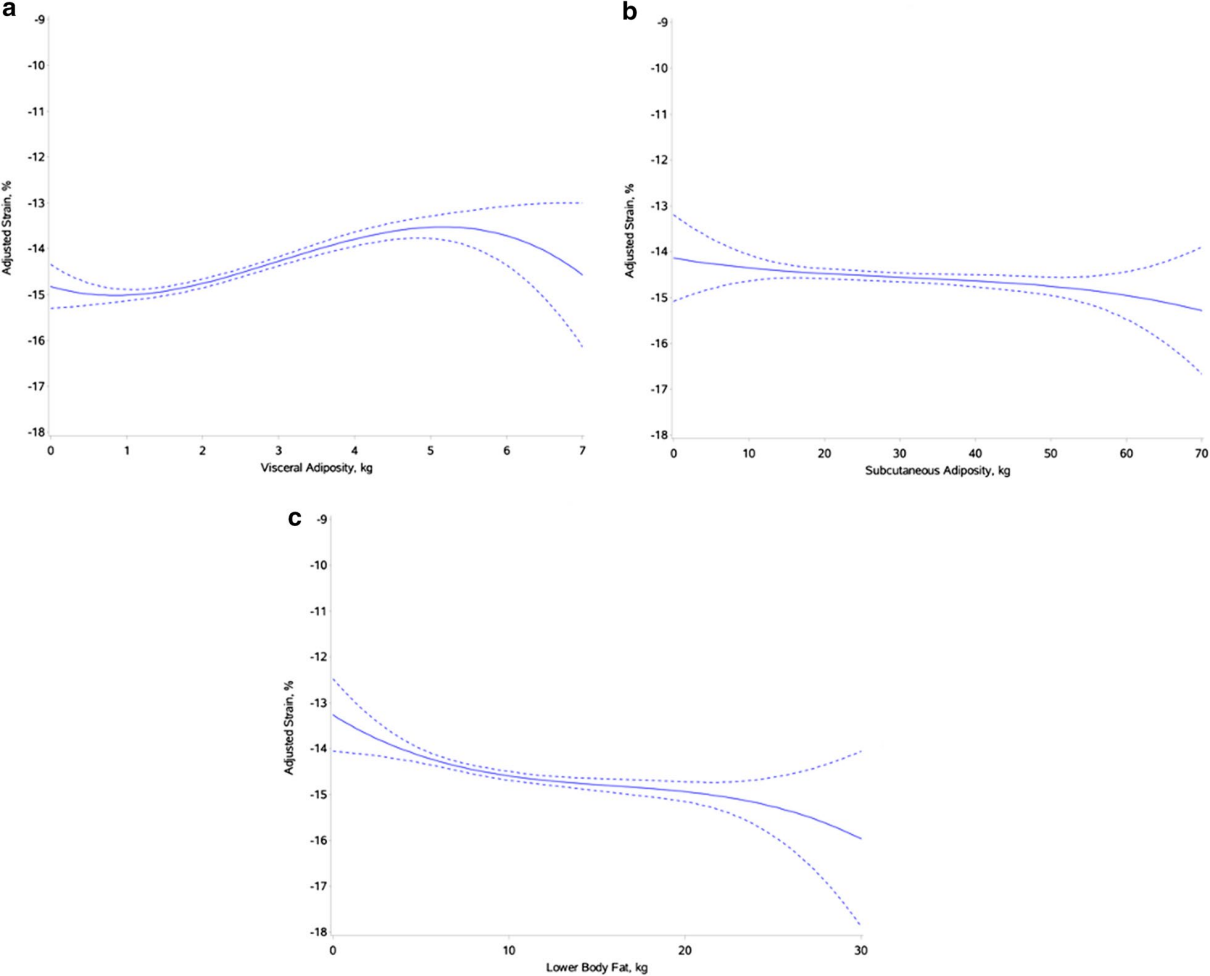
Restricted cubic splines of visceral adiposity (**a**), subcutaneous adiposity (**b**), and lower-body fat (**c**), and adjusted peak circumferential strain. Each model adjusts for cardiovascular disease (CVD) risk factors, cardiorespiratory fitness (CRF), and left ventricular (LV) parameters (LV mass, LV ejection fraction (LVEF)

Multivariable adjusted cross-sectional associations of regional fat depots with E_cc_ as observed using restricted cubic splines are shown in Fig. 1. In adjusted analysis, higher amount of VAT was significantly associated with higher E_cc_ suggesting greater impairment in LV strain after adjustment for traditional CVD risk factors (β: 0.13, P = 0.002; Table 2, Model 1). The association remained significant after further adjustment for CRF (β: 0.09, P = 0.042; Table 2, Model 2), LV parameters (β: 0.15, P = 0.001; Table 2, Model 3), and other fat depots (β: 0.19, P = 0.002; Table 2, Model 4). Among other regional body fat depots, SAT was not associated with E_cc_ in adjusted analysis. Higher LBF was associated with lower E_cc_ suggesting more favorable LV contractility after adjustment for CVD risk factors, CRF, and LV parameters (β: − 0.12, P = 0.005; Table 2, Model 3). However, this association was not significant after adjusting for VAT and SAT (β: -0.18, P = 0.054; Table 2, Model 4). In sensitivity analysis replacing total lean mass with ALM or ALM index in the most adjusted model, the associations of VAT, SAT and LBF with E_cc_ were consistent with that observed in the primary analysis (Additional file 1: Table S1). Furthermore, additional adjustment for MVPA did not modify the observed associations of VAT, SAT, and LBF with E_cc_ (Additional file 1: Table S1).

**Table 2:** Multivariable-adjusted associations between VAT, SAT, and LBF with left ventricular peak systolic strain

	Model 1		Model 2		Model 3		Model 4	
	Std. beta	P-value	Std. beta	P-value	Std. beta	P-value	Std. beta	P-value
VAT	0.13	0.002	0.09	0.042	0.15	0.001	0.19	0.002
SAT	− 0.04	0.412	− 0.11	0.02	− 0.03	0.455	0.01	0.912
LBF	− 0.11	0.011	− 0.17	0.001	− 0.12	0.005	− 0.18	0.054

Separate multivariable adjusted linear regression models for VAT, SAT, LBF (independent variables) respectively, and Ecc (dependent variable). Model 1 adjusts for CVD risk factors including age, sex, race, smoking, fasting glucose, diabetes status, hypertension status, systolic blood pressure, and lean body mass. Model 2 adjusts for CVD risk factors and CRF. Model 3 adjusts for CVD risk factors, CRF, and LV parameters (LV mass, LVEF). Model 4 adjusts for CVD risk factors, CRF, and LV parameters (LV mass, LVEF), and includes VAT, SAT, and LBF in the same model. Standardized beta estimate represents change in the outcome of interest per 1-SD increase in the primary exposure variable keeping other covariates fixed. *VAT* visceral adipose tissue, *SAT* subcutaneous adipose tissue, *LBF* lower-body fat, peak systolic circumferential strain (Ecc), left ventricular systolic strain, *CVD* cardiovascular disease, *CRF* cardiorespiratory fitness, *LV* left ventricular, *EF* ejection fraction

In stratified analysis across BMI based subgroups (obese vs. non-obese), the association between higher amount of VAT and impairment in E_cc_ was stronger in non-obese vs. obese participants. In contrast, the association between higher amounts of LBF and favorable E_cc_ was more evident in obese but not in non-obese participants. SAT was not associated with E_cc_ in adjusted analysis in both non-obese and obese subgroups (Table 3).

**Table 3:** Multivariable-adjusted associations of VAT, SAT, and LBF with left ventricular peak systolic strain stratified by body mass index

	Nonobese		Obese	
	Std. beta	P-value	Std. beta	P-value
VAT	0.16	0.056	0.01	0.849
SAT	0.16	0.205	− 0.10	0.377
LBF	-0.16	0.117	− 0.22	0.058

Multivariable adjusted linear regression models for VAT, SAT, LBF (independent variables) and Ecc (dependent variable) stratified by BMI. Nonobese defined as BMI ≤ 30 m/kg^2^. Obese defined as > 30 kg/m^2^. Model adjusts for age, sex, race, smoking, fasting glucose, diabetes status, hypertension status, systolic blood pressure, lean body mass, CRF, and LV parameters (LV mass, LVEF), and includes VAT, SAT, and LBF in the same model

Standardized beta estimate represents change in the outcome of interest per 1-SD increase in the primary exposure variable keeping other covariates fixed. *VAT* visceral adipose tissue, *SAT* subcutaneous adipose tissue, *LBF* lower-body fat, peak systolic circumferential strain (Ecc), left ventricular systolic strain; *BMI* body mass index, *CVD* cardiovascular disease, *CRF* cardiorespiratory fitness, *LV* left ventricular, *EF* ejection fraction

### Association of CRF, MVPA, and lean body mass with peak circumferential strain

Higher CRF was significantly associated with lower E_cc_ suggesting more favorable contractility independent of CVD risk factors, other echo parameters, regional fat depots, and overall lean mass or ALM (Table 4) (Additional file 1: Table S1). In contrast, MVPA was not associated with E_cc_ after accounting for other confounders including CRF. Measures of ALM and ALM index were also not associated with E_cc_ in the most adjusted model (Table 4).

**Table 4:** Multivariable-adjusted associations between appendicular lean mass, physical activity and left ventricular peak systolic strain

	Std. beta	P-value
ALM^a^	− 0.05	0.380
ALM index^b^	− 0.01	0.971
MVPA^c^	0.01	0.897
CRF^c^	− 0.15	< .001

Standardized beta estimate represents change in the outcome of interest per 1-SD increase in the primary exposure variable keeping other covariates fixed. *ALM* appendicular lean mass, *CRF* cardiorespiratory fitness, *MVPA* moderate to vigorous physical activity

^a^Multivariable adjusted linear regression model with Ecc as an outcome and following covariates: age, sex, race, smoking, fasting glucose, diabetes status, hypertension status, systolic blood pressure, ALM, CRF, LV mass, LVEF, VAT, SAT, and LBF

^b^Multivariable adjusted linear regression model with Ecc as an outcome and following covariates: age, sex, race, smoking, fasting glucose, diabetes status, hypertension status, systolic blood pressure, ALM index, CRF, LV mass, LVEF, VAT, SAT, and LBF

^c^Multivariable adjusted linear regression model with Ecc as an outcome and following covariates: age, sex, race, smoking, fasting glucose, diabetes status, hypertension status, systolic blood pressure, total body lean mass index, CRF, LV mass, LVEF, VAT, SAT, LBF, and MVPA

## Discussion

**Fig. 2:**
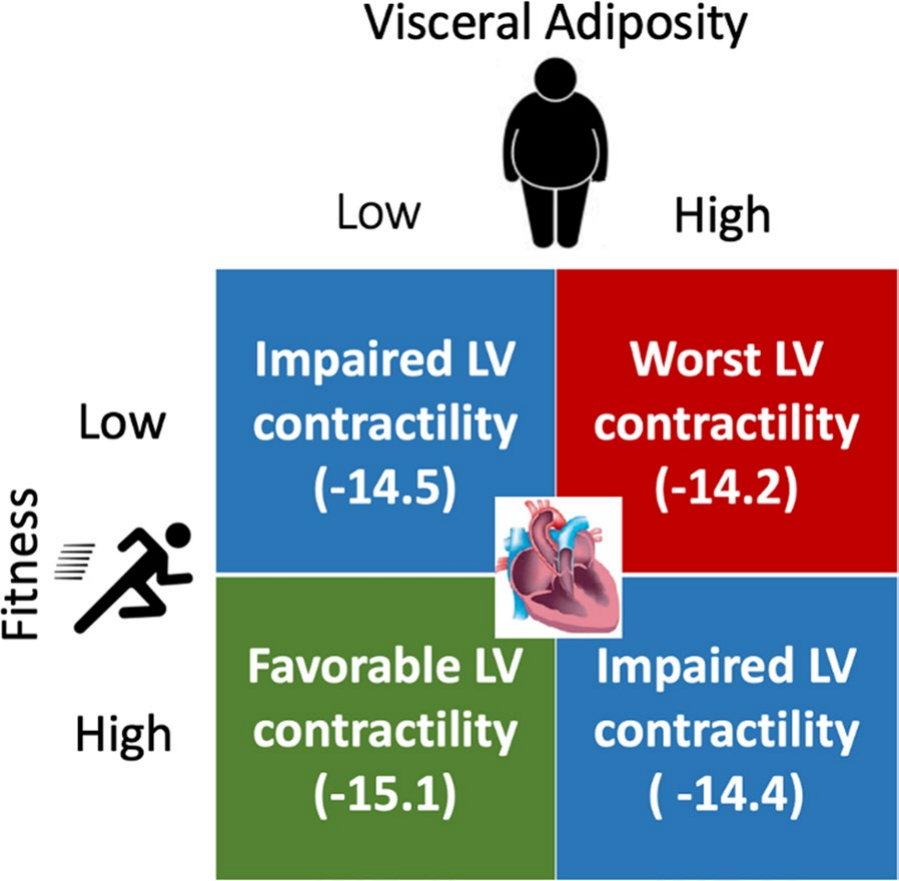
Association of varying visceral adiposity (VAT) (high/low) and CRF (high/low) levels with LV contractility. The cohort is divided into 4 groups based on high and low CRF and VAT by race and sex specific medians. Mean peak systolic circumferential strain (E_cc_) of each group is reported

In this cohort of individuals free of CVD, we made several important observations. First, higher amounts of VAT were associated with greater impairment in LV contractility, as assessed by E_cc_, independent of traditional CVD risk factors, CRF, LV parameters, and other adipose tissue depots. The association between higher amounts of VAT and greater impairment in LV contractility was more prominent among non-obese individuals as compared with obese individuals. Second, higher LBF was associated with a trend towards more favorable LV contractility while SAT was not associated with LV contractility. Third, higher CRF was associated with more favorable LV contractility, independent of other risk factors, LV parameters, and regional adipose tissue depots. Finally, total lean body mass as well as ALM were not associated with measures of LV contractility after adjustment for other potential confounders. Our findings highlight the independent contributions of VAT and CRF to LV subclinical systolic dysfunction (Fig. 2), an intermediate phenotype associated with greater risk of HF.

Previous studies have characterized the abnormal cardiac remodeling patterns associated with higher VAT. Specifically, investigators have reported a cross-sectional association between high VAT and increased LV concentricity (higher LV mass, lower LVEDV), independent of CVD risk factors and BMI [5, 8, 9]. In contrast, studies have not demonstrated an independent association between VAT and systolic dysfunction as measured by LVEF [5, 23]. LVEF is a crude measure of LV systolic function and may not be sensitive enough to capture subtle abnormalities in LV contractility in otherwise healthy individuals. Consistent with this notion, prior studies have demonstrated a significant inverse association between VAT and LV contractility as assessed by global longitudinal strain [24, 25]. However, these studies did not account for differences in other regional adiposity depots and CRF, which may be important confounders in the observed associations. Findings from the present study add to the existing literature demonstrating a significant association between LV strain and abnormalities in LV contractility independent of CRF and other regional adiposity depots.

The location of excess adiposity may influence the cardiac remodeling pattern that occurs in obese individuals. While VAT is implicated in the development of concentric LV remodeling, excess LBF has been more strongly associated with eccentric LV remodeling [9]. In contrast, SAT has not been consistently associated with abnormal LV remodeling in previous studies [8, 9]. Consistent with prior observations, SAT was not associated with LV contractility in the adjusted models in the present study. However, we observed an association between higher amounts of LBF and more favorable LV contractility. Several factors may underlie the observed association between LBF and favorable LV contractility. Previous studies have demonstrated a significant association between higher LBF and lower burden of cardiovascular risk factors, less concentric remodeling, improved systemic vascular resistance, and lower rate of cardiovascular disease [9, 26, 27]. Also, LBF may contribute to a more favorable cardiometabolic profile by serving as a metabolic sink, absorbing excess circulating lipids thereby protecting other tissues from the harmful downstream consequences of ectopic fat deposition [28, 29]. Consistent with this notion, we observed a more favorable association between LBF and LV contractility in obese individuals but not in non-obese individuals.

The mechanisms underlying the observed association between higher VAT and worse LV strain are not well established. VAT is epidemiologically linked to CVD risk factors such as hyperlipidemia, hypertension, and diabetes [30, 31], and may lead to abnormal cardiac remodeling and LV contractility through the excess burden of risk factors [5, 32]. Consistently, studies have observed that risk of HF associated with VAT is driven by greater burden of cardiometabolic risk factors [5, 33]. It is noteworthy that a more independent association between VAT and risk of HF has been reported, specifically with HF with preserved LVEF (HFpEF) [12]. Findings from our study suggest that the higher risk of HF, particularly HFpEF, in patients with higher amounts of VAT may be related to impairment in myocardial contractility independent of traditional CVD risk factors. This is particularly relevant since studies have implicated LV strain impairment in the development of HFpEF [34]. We also observed that the association between higher VAT and worse LV contractility was more pronounced among participants in the normal to overweight BMI range. This highlights the potential adverse cardiac effects of higher visceral adiposity in normal to overweight individuals. Consistent with our observation, prior studies have linked higher VAT and central adiposity to adverse cardiometabolic profile, greater arterial stiffness, and greater CVD risk in normal to overweight individuals [35–37]. VAT may mediate myocardial dysfunction through a number of mechanisms. VAT activates macrophages leading to the secretion of cytokines such as interleukin-6 and tumor necrosis which may potentiate inflammation and fibrosis of the myocardium [38]. Locally, the presence of VAT induces changes in cardiac tissue that lead to myocardial fibrosis, cardiomyocyte hypertrophy, and macrophage infiltration, all of which may contribute to abnormal cardiac remodeling patterns and dysfunction in myocardial contractility.

We observed that higher CRF levels were associated with more favorable LV contractility independent of other risk factors as well as regional adiposity burden. Prior studies have observed an inverse association between CRF and HF risk, independent of CVD risk factor burden and BMI [39–43]. Furthermore, low CRF has been associated with abnormal cardiac remodeling patterns characterized by higher LV filling pressure, diastolic dysfunction, and impairment in E_cc_ [10, 11, 16]. It is noteworthy that prior analyses linking CRF to abnormalities in cardiac structure and function and downstream HF risk did not account for differences in regional adiposity burden. The present study suggests the positive association between high CRF and favorable cardiac remodeling, may be independent of regional adiposity burden.

Our study findings have important clinical implications. The independent associations of higher VAT and lower CRF with subclinical abnormalities in LV contractility highlight their potential role as modifiable targets for HF prevention [44, 45]. Observational studies of individuals who have undergone bariatric surgery suggest that substantial weight loss can reverse pathological cardiac remodeling and reduce risk of incident HF[46, 47]. More recently, findings from the Look AHEAD trial have demonstrated that greater loss of overall and central adiposity with lifestyle interventions is associated with lower risk of HF [48]. Furthermore, temporal improvement in CRF has been associated with improved systolic and diastolic parameters [11] and lower risk of HF [7]. Despite these promising observations, interventions targeting non-specific weight loss and improvement in CRF have not demonstrably been shown to reduce HF risk [43, 49]. Future studies are needed to evaluate if preventive strategies aimed at lowering VAT and optimizing CRF can favorably modify LV structure and function and mitigate the downstream risk of HF.

### Limitations

Our study has limitations. First, our study is cross-sectional and therefore does not establish a causal relationship between VAT, CRF, and subclinical systolic dysfunction. Second, our study was observational, rendering it susceptible to residual confounding. Third, our findings may not be generalizable to individuals with established CVD, as LV morphology and function may be different compared to our study population. Fourth, there was a lack of data on right ventricular parameters and diastolic function, both of which may represent intermediate phenotypes along the progression to clinical HF [13, 50]. This precluded inclusion of these parameters into the analysis. Fifth, the present study did not have available data on muscular strength, which is associated with CRF and is implicated in the development and progression of HF [51, 52]. However, we observed no associations between overall lean mass and ALM, with LV contractility. Furthermore, the cross-sectional associations between VAT, CRF and E_cc_ were also independent of total lean mass and ALM. Sixth, DEXA derived estimates of VAT were used in the present analysis, as magnetic resonance imaging, a superior modality for quantifying VAT, was not available. Seventh, there was moderate degree of variability in our E_cc_ measures based on correlation and Bland Altman plot statistics highlighting the need for validation of the observed results in other cohorts. Finally, due to a limited number of incident HF events in the DHS cohort, the longitudinal associations between E_cc_ and regional adiposity with incident HF could not be assessed.

In conclusion, in a multiethnic cohort of adults without CVD, higher amounts of VAT were associated with myocardial dysfunction as measured by E_cc_, independent of traditional CVD risk factors, other echocardiographic parameters, and CRF. Furthermore, lower CRF was associated with impairment in E_cc_ independent of CVD risk factors and regional adiposity burden. These findings suggest VAT and CRF may mediate pathological cardiac remodeling through distinct processes. More studies are needed to investigate VAT and CRF as potential targets for HF prevention, and elucidate the interplay between VAT, CRF, and BMI in the development of HF.

## Supplementary Information

**Additional file 1: Table S1.** Multivariable-Adjusted Associations between VAT, SAT, and LBF with Left Ventricular Peak Systolic Strain, with Adjustment for ALM and ALM index.** Figure S1.** Bland-Altman Plot (A) and Line Regression (B) of Peak Circumferential Strain Measurements. The Bland Altman plot displays the mean difference between the 2 measurements for each participant plotted on the Y axis. The mean of the 2 measurements is plotted on the X axis. The linear regression displays the first strain measurement on the X-axis and the second strain measurement on the Y-axis.

## Data Availability

The data that support the findings of this study are available in the Dallas Heart Study Cohort. The data is not publicly available; however, it can be made available from the authors upon request to the Dallas Heart Study Cohort panel.

## Abbreviations

ALM: Appendicular lean mass
BMI: Body mass index
CMR: Cardiovascular magnetic resonance
CRF: Cardiorespiratory fitness
CVD: Cardiovascular disease
DEXA: Dual-energy X-ray absorptiometry
DHS: Dallas Heart Study
E_cc_: Peak systolic circumferential strain
ECG: Electrocardiogram
EDV: End-diastolic volume
HF: Heart failure
HFpEF: Heart failure with preserved ejection fraction
LBF: Lower-body fat
LV: Left ventricle/left ventricular
LVEDV: Left ventricular end-diastolic volume
LVEF: Left ventricular ejection fraction
LVESV: Left ventricular end-systolic volume
MVPA: Moderate to vigorous physical activity
VAT: Visceral adiposity
VO_2_: Oxygen uptake
VO_2max_: Peak oxygen uptake
