# Supplementary material for: Regional adiposity, cardiorespiratory fitness, and left ventricular strain: an analysis from the Dallas Heart Study
Source: J Cardiovasc Magn Reson. 2021 Jun 14;23:78. doi: 10.1186/s12968-021-00757-w (PMC8201708; doi:10.1186/s12968-021-00757-w)

**Additional MATERIAL**

**AdditionalTable 1**. Multivariable-Adjusted Associations between VAT, SAT, and LBF with Left Ventricular Peak Systolic Strain, with Adjustment for ALM and ALM index.

|  | **Model 4** | | **Model 5** | | **Model 6** | | **Model 7** | |
| --- | --- | --- | --- | --- | --- | --- | --- | --- |
|  | Std. Beta | P-value | Std. Beta | P-value | Std. Beta | P-value | Std. Beta | P-value |
| VAT | 0.19 | 0.002 | 0.19 | 0.002 | 0.19 | 0.002 | 0.17 | 0.008 |
| SAT | 0.01 | 0.912 | 0.01 | 0.936 | -0.01 | 0.926 | 0.05 | 0.683 |
| LBF | -0.18 | 0.054 | -0.17 | 0.078 | -0.17 | 0.075 | -0.22 | 0.031 |
| Separate multivariable adjusted linear regression models for VAT, SAT, LBF (independent variables) respectively, and E_cc_ (dependent variable). Model 4 adjusts for CVD risk factors, total lean body mass, CRF, and LV parameters (LV mass, LVEF), and includes VAT, SAT, and LBF in the same model. Model 5: Model 4 except ALM replaces total lean body mass. Model 6: Model 4, except ALM index replaces total lean body mass. Model 7: Model 4 + MVPA. Standardized beta estimate represents change in the outcome of interest per 1-SD increase in the primary exposure variable keeping other covariates fixed. VAT, visceral adipose tissue; SAT, subcutaneous adipose tissue; LBF, lower-body fat; CVD, cardiovascular disease; CRF cardiorespiratory fitness; LV, left ventricular; LVEF, left ventricular ejection fraction; ALM, appendicular lean mass; MVPA, moderate to vigorous physical activity. | | | | | | | | |

**Additional Figure 1.** Bland-Altman Plot (A) and Line Regression (B) of Peak Circumferential Strain Measurements. The Bland Altman plot displays the mean difference between the 2 measurements for each participant plotted on the Y axis. The mean of the 2 measurements is plotted on the X axis. The linear regression displays the first strain measurement on the X-axis and the second strain measurement on the Y-axis.


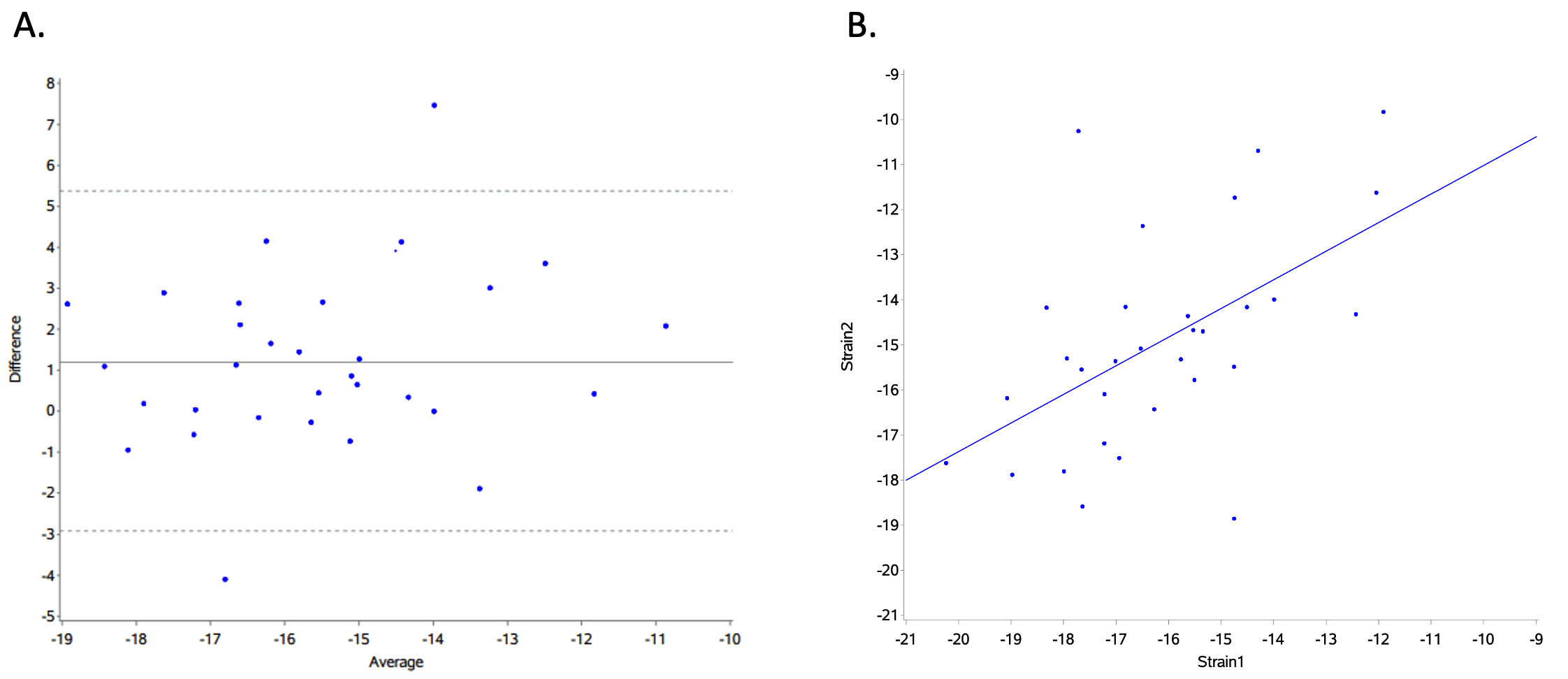

Supplement: Supplementary file 1 — Additional file 1: Table S1. Multivariable-Adjusted Associations between VAT, SAT, and LBF with Left Ventricular Peak Systolic Strain, with Adjustment for ALM and ALM index. Figure S1. Bland-Altman Plot (A) and Line Regression (B) of Peak Circumferential Strain Measurements. The Bland Altman plot displays the mean difference between the 2 measurements for each participant plotted on the Y axis. The mean of the 2 measurements is plotted on the X axis. The linear regression displays the first strain measurement on the X-axis and the second strain measurement on the Y-axis. [file 12968_2021_757_MOESM1_ESM.docx]
